# Determination of natural radioactivity in products of animals fed with grass: A case study for Kars Region, Turkey

**DOI:** 10.1038/s41598-020-63845-4

**Published:** 2020-04-24

**Authors:** Gülçin Bilgici Cengiz

**Affiliations:** Department of Physics, Faculty of Arts and Sciences, Kafkas University, 36100 Kars, Turkey

**Keywords:** Environmental sciences, Natural hazards, Chemistry, Physics

## Abstract

In this research, the activity concentrations of ^40^ K, ^232^Th and ^226^Ra in 41 grass samples collected from Kars region, Turkey, were determined using gamma ray spectrometry. Natural radioactivity concentrations in animal food products were calculated based on activity concentrations of these radionuclides in pasture-grass samples and dry-grass consumption of animals. The average annual effective dose from these radionuclides for local consumers due to indirect ingestion of cow milk, sheep milk, poultry, mutton and beef consumption have been calculated as 9.01, 0.24, 1.76, 0.38 and 5.25 µSv y^-1^, respectively. Furthermore, the calculated average annual effective dose values for adults are within the values found in other countries worldwide. These results show that animal products can be safe for human consumption in terms of radiation exposure due to the natural radionuclides studied.

## Introduction

Determination of environmental radioactivity levels is essential to estimate the radiation levels to which people are directly or indirectly exposed. Therefore, environmental samples have been widely examined by researchers in order to determine the natural radioactivity levels. In the literature, there are many studies aiming to determine the levels of natural radiation in the soil and the radiation hazard indices that can arise from the natural radionuclides in soil^1–5^. The radiation levels in food stuff are of interest to researchers because ingestion is one of the most widespread way in which radionuclides immigrate to living beings^6–9^. Santos et al. reported that the estimated annual effective dose due to the intake of vegetables and their derived products by the adult inhabitants of Rio de Janeiro City with the natural radionuclides (^232^Th, ^238^U, ^210^Pb, ^226^Ra and ^228^Ra), reached 14.5 μSv. They also found that when the water and milk data were considered, the dose value increases to 29 μSv^10^. Naturally occurring radionuclides such as ^40^ K, ^232^Th, and ^226^Ra find their way to reach the food chain from soil and air to plants, and from plants to animals and also to human beings^11^. For this reason, it is useful to monitor radiation levels in animal feed such as grass or fodder plants. Natural radionuclides found in animal products consumed by humans such as beef, chicken, milk and eggs can be transferred to humans through food chain^12^. In a study in Iran conducted by Sarayegord *et al*. (2009), the average activity concentrations for ^40 ^K (31.0 ± 6.1 Bq.kg^−1^) in examined milk samples was used to calculate the effective dose of milk in adults as 14 µSv.year^−1^ ^13^. As a result of the study conducted in Egypt; the irradiation risk of human health was evaluated owing to indirect ingestion of the beef, milk, poultry and egg, the annual effective dose of the radionuclides for the local consumer was found to be as 2.7, 14.0, 0.1, and 0.14 µSv, respectively^14^.

The aim of this study is to theoretically determine the natural radionuclide levels in animal products obtained from animals fed with these dry-grass by using the natural radioactivity levels determined in the grasses grown in Kars region. In addition, the results of this study have been used to describe the annual effective dose to the local population due to ingestion of natural radionuclides in animal products.

## Materials and Methods

### Sample collection and preparation

Since pasture plants are rich in protein, minerals and vitamins, they can meet the nutritional needs of all grass-eating animals. Pasture plants can be given as fresh, dry and silage to animals and can be grown for grazing^15^. Grazing of various animal species in the pastures is the most economical and correct way^16^. If the pastures are not used according to a certain system, they will deteriorate immediately. In order not to deteriorate the pasture structure and get the maximum yield from the pasture, it is necessary to graze with animal species suitable for pasture type during grazing season. The grazing pattern of each animal species is different. They also have the characteristics of choosing the grasses that animals graze. Cattle eat grasses with their tongues at a height of 3 to 4 cm. When cattle first go to the pasture, they do not cause much damage to the pasture since they first eat the leaf ends of the plants^17^. Sheep and geese pick up plants very close to the soil surface. In other words, the amount of stubble remaining is very low^18^. If grazing is done frequently and with more animals, the pasture will be damaged. In the breeding of pastures which are disturbed by sheep and horses, it is also struggled by grazing with cattle^16,18^. Considering the grazing habits of the animals grazing in the pastures, the plants collected in our study were divided into three sections^19^.

**Figure 1:**
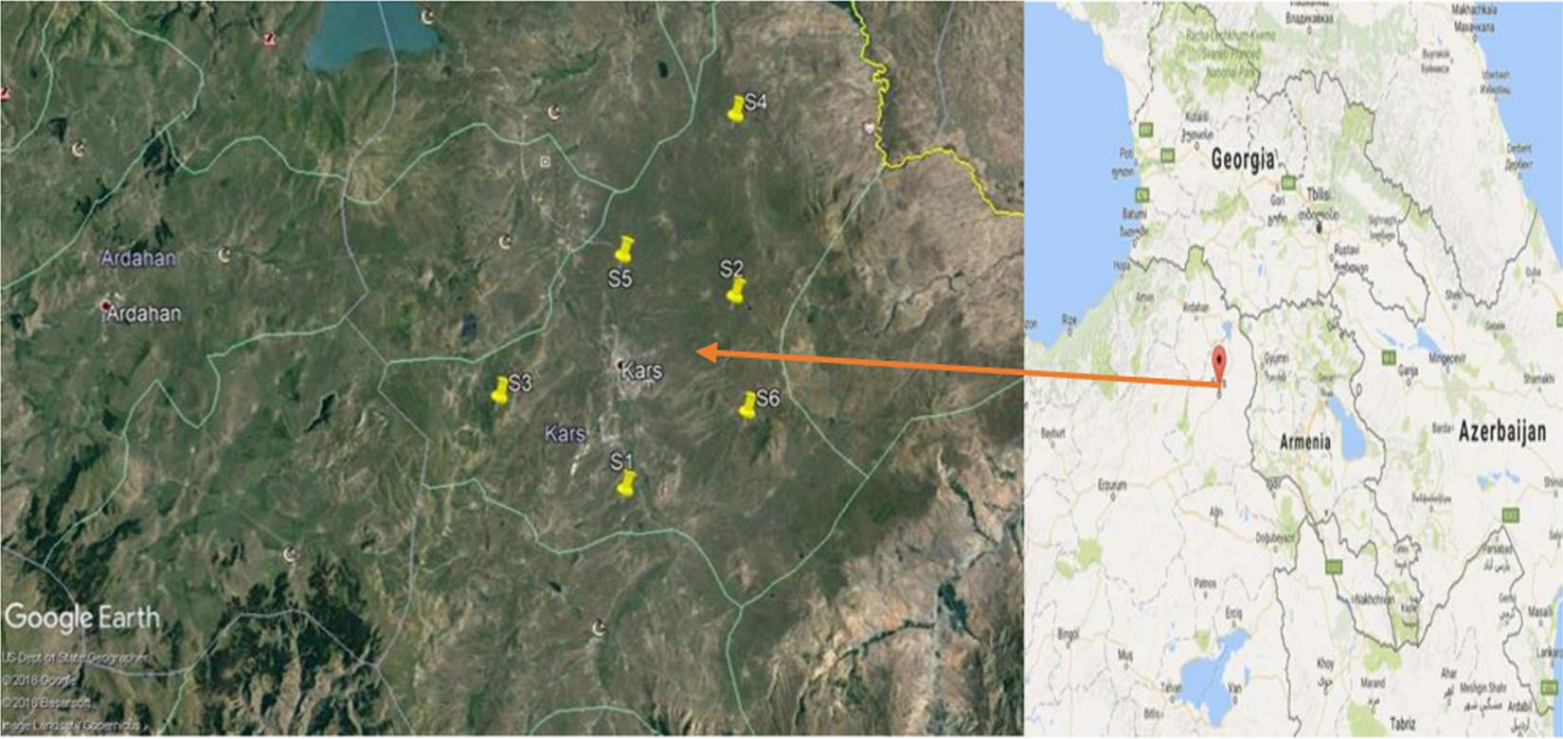
Satellite map of sampling stations where pasture samples are collected.

Activity concentrations of natural radionuclides were determined by examining a total of 41 grass plant samples taken from 6 different stations in Kars province. Kars is located in the northeastern part of the country and shares part of its border with the Republic of Armenia. Geographical coordinates of Kars are 40° 25’ 0″ north latitude and 43° 4′ 59″ east longitudes and have an average altitude of 1768 meters above sea level. Its surface area is 18557 km² and the total population is 288878 as of 2018. The province’s economy is based on agriculture and animal husbandry because of the excess of pastures and meadows in Kars. Due to the proximity of the Metzamor Nuclear Facility in Armenia to the grasslands, the location of the sampling area of the selected grass was measured by a GPS instrument and the coordinates of the sampling sites were shown on the map with the Google Earth Map application (Fig. 1).

Approximately 5 kg of grassland plants were collected. After removal of any trace of soil, the plant samples were divided in three part as root, stem and leaves and dried in an oven at 105 °C for 12 hours. The dried plant samples were powdered and then ashed into a white ash at 425 °C in an electrical oven where the temperature gradually increased. Depending on the approximate grass samples presence, 0.07–0.09 kg of ashed plant sample was prepared and was transferred to cylindrical plastic containers with a diameter of 6 cm and a height of 5 cm. Containers of similar size and shape were used to maximize counting efficiency and accuracy and to minimize self-absorption for this particular geometry. Then samples were weighed and hermetically sealed to prevent the escape of radon gas. Samples were kept for 45 days to obtain a secular balance between ^226^Ra (^238^U daughter) and ^232^Th daughter before the measurements^20^.

### Activity determination

The activity of each sample was counted for a period of 86000 s using NaI(Tl) detector based on gamma-ray spectrometer system. The spectrum was analyzed using a PC (Personal Computer) based an MCA (Multi Channel Analyzer) system and Maestro software. The energy calibration and the relative efficiency calibration of the gamma spectrometer were performed using the certified reference material IAEA-375 Soil (originating from the area affected by the Chernobyl accident)^21^. For determining the activity concentrations in the soil samples and pasture-grass samples, suitable photopeaks at several energies were taken into account and the appropriate area (ROI) regions were selected for each peak. The activity concentrations of ^40^K was evaluated from the 1460.8 keV gamma line. ^226^Ra concentration was found out by measuring the 609.3, 1120.3 and 1764.5 keV gamma-rays from ^214^Bi. Similarly, 583 keV and 2614.5 keV gamma-rays from ^208^Tl were used to indicate the activity concentration of ^232^Th. The net count rate under the most significant photo peaks of all radionuclides daughter peaks were determined by subtracting the background spectrum corresponding to the same count time. Afterwards the activity of radionuclide the background subtracted area, is calculated from the significant gamma ray energies^20^.

### Annual effective dose from animal products

As calculated in similar studies the activity concentrations of ^226^Ra, ^232^Th and ^40^K in each portion of pasture samples, daily dry matter feed intake rates and transition coefficients for animal products were used to calculate the activity concentrations of these mentioned radionuclides in some animal products (Eq. 1)^14^.

1 $${A}_{i}={A}_{j}x{Q}_{j}x{R}_{i}$$

where, A_i_ is the calculated activity concentration in animal products, beef, milk and poultry in Bq kg^−1^. A_j_ is the average radionuclide concentration in grass. Q_j_ is the average amount of dry-grass per day consumed by cattle, sheep and poultry (kg day^−1^), and R_i_ is the fraction of the animal’s daily intake by ingestion transferred to animal products (day kg^−1^ or day l^−1^)^22,23^.

Calculation of the annual effective dose due to internal irradiation caused by the radionuclides present in foods (^226^Ra, ^232^Th and ^40^K) is important for the assessment of the possible radiological risk for human health. Considering the IAEA’s recommendations, radionuclide levels should be investigated separately in each of nutrients people consume in their daily life^24^. The mean ingestion dose should be calculated by considering the average activity levels of the natural radionuclides in each food. According to Till and Moore (1988), the annual effective dose from natural consumption of food is calculated by considering the radionuclide activity measured (Bq kg^−1^) in food, the amount of food consumed by people per year (kg y^−1^) and the effective dose conversion factor of each radionuclide^25^. Hence, the average annual effective dose for human due to ingested animal products could be calculated using Eq. (2).

2 $$Annual\,Effective\,dose,\,D\,(mSv{y}^{-1})=({A}_{Ra}xD{C}_{Ra}+{A}_{Th}xD{C}_{Th}+{A}_{K}xD{C}_{K})U$$

where, A_Ra_, A_Th_ and A_K_ are activity concentration of ^226^Ra, ^232^Th and ^40^K in Bq kg^−1^, respectively. The dose conversion factor for ^226^Ra, ^232^Th and ^40^K are DC_Ra_ (0.28 µSv Bq^−1^), DC_Th_ (0.23 µSv Bq^−1^ and DC_K_ (0.0062 µSvBq^−1^), respectively^26^. U (kg y^−1^) is the annual consumption rate of animal products by people.

## Results and Discussions

The of ^226^Ra, ^232^Th and ^40^K radioactivity concentrations in Bq kg^−1^ (dry weight) in different parts of pasture-grass samples are presented in Table 1. The mean activity concentrations of ^226^Ra, ^232^Th and ^40^K in the pasture-grass samples of Kars region ranged from from 21.8 ± 6.2 Bqkg^−1^ to 50.1 ± 13.8 Bqkg^−1^ with an average of 31.4 ± 8.4 Bqkg^−1^, 51.7 ± 12.2 Bq Kg^−1^ to 129.7 ± 23.0 Bqkg^−1^ with an average of 92.7 ± 17.8 Bqkg^−1^ and 309.7 ± 33.4 Bqkg^−1^ to 800.3 ± 84.7 Bqkg^−1^ with an average value of 606.3 ± 61.7 Bqkg^−1^, respectively. The daily dry matter feed intake rates of farm animals and transition coefficients for animal products are listed in Tables 2 and 3, respectively.

**Table 1:** Activity concentration (Bq·kg^–1^, dry weight) of the radionuclides in each part of pasture grass samples.

Sample Name	Activity Concentrations (Bqkg^−1^)								
	Lower Part			Stem Part			Leave Part		
	^226^Ra	^232^Th	^40^K	^226^Ra	^232^Th	^40^K	^226^Ra	^232^Th	^40^K
S1	40.3 ± 8.5	67.5 ± 12.6	570.2 ± 51.6	34.8 ± 9.6	106.7 ± 17.5	744.3 ± 64.4	31.0 ± 8.3	105.0 ± 19.2	800.3 ± 84.7
S2	34.1 ± 8.3	87.2 ± 17.7	402.4 ± 50.0	36.7 ± 8.2	129.7 ± 23.0	794.9 ± 76.4	33.8 ± 9.0	89.7 ± 21.2	798.9 ± 79.4
S3	24.0 ± 6.0	51.7 ± 12.2	605.3 ± 60.4	40.0 ± 10.8	107.1 ± 18.2	702.8 ± 62.1	27.2 ± 6.0	100.9 ± 16.8	732.8 ± 62.1
S4	21.8 ± 6.2	58.8 ± 18.8	550.8 ± 53.1	27.3 ± 8.4	106.8 ± 19.4	664.1 ± 69.5	22.3 ± 6.5	84.4 ± 15.3	784.0 ± 72.6
S5	50.1 ± 13.8	79.8 ± 15.1	510.6 ± 54.6	25.5 ± 8.8	97.0 ± 18.5	309.7 ± 33.5	24.7 ± 6.4	114.9 ± 23.0	424.8 ± 60.5
S6	29.1 ± 7.9	93.1 ± 19.7	399.1 ± 55.3	23.4 ± 8.4	110.9 ± 22.3	510.7 ± 52.3	39.8 ± 9.8	78.7 ± 18.5	608.7 ± 70.7
Mean	33.2 ± 8.5	73.0 ± 15.2	506.4 ± 54.2	31.3 ± 9.3	109.7 ± 19.3	621.1 ± 59.7	29.8 ± 7.7	95.7 ± 17.6	691.1 ± 71.6

**Table 2:** Daily dry matter feed intake rates of farm animals^15–17^.

Animal Types	Dry matter (kgd^−1^)
Dairy cows	16.1
Dairy sheep	1.3
Cattle	7.2
Lamb	1.1
Laying poultry	0.1
Poultry	0.07

**Table 3:** Transfer factors for animal products (day kg^−1^ or day l^−1^)^22^.

Animal Products	Transfer factor (day kg^−1^ or day l^−1^)		
	^226^Ra	^232^Th	^40^K
Beef	9.0 × 10^−4^	4.0 × 10^−5^	2.0 × 10^−2^
Poultry	3.0 × 10^−2^	6.0 × 10^−3^	4.0 × 10^−1^
Milk	1.3 × 10^−3^	5.0 × 10^−6^	7.2 × 10^−3^

The estimating values for the daily dry matter feed intakes of farm animals are given in Table 2 and transfer factors of ^226^Ra, ^232^Th and ^40^K radionuclides from dry matter feed to animal products are shown in the Table 3.

Based on the concentration of ^226^Ra, ^232^Th and ^40^K radionuclides in each portion of the pasture and the amount of grass eaten by the animals, it was found that the concentrations of ^226^Ra, ^232^Th and ^40^K radionuclides calculated in each animal product would be different. Table 4 shows calculated activities of ^226^Ra, ^232^Th and ^40^K radionuclides in animal products in Bqkg^−1^. The range of average ^40^K concentration was computed to be 5.8–99.6 Bq kg^−1^. Mutton, sheep milk and poultry are supplied from farm animals fed with stem and root parts of the grass from small ruminants and poultry, respectively. It was estimated that the concentration of ^40^K in beef meat and cow milk obtained from bovine animals fed with the leaf part of the grass was higher than the concentration of ^40^K in mutton, sheep milk and poultry. This is because ^40^K accumulates mostly in the stem and leaf part of the grass where cattle are fed^20^.

**Table 4:** The concentration of ^226^Ra, ^232^Th and ^40^K (Bq kg^−1^) in animal products.

Products	Activity Concentration (Bq kg^−1^)		
	^226^Ra	^232^Th	^40^K
Beef Mutton Poultry Cow Milk Sheep Milk	1.9 × 10^−1^	2.8 × 10^−2^	99.6
	3.1 × 10^−2^	5.0 × 10^−3^	13.7
	7.0 × 10^−2^	3.1 × 10^−2^	14.2
	6.2 × 10^−1^	8.0 × 10^−3^	80.2
	5.2 × 10^−2^	7.1 × 10^−4^	5.8

The concentration of ^226^Ra in animal product was ranged from 3.1 × 10^−2^ Bq kg^−1^ (mutton) to 6.2 × 10^−1^ Bq kg^−1^ (cow milk). As can be seen from Table 4, the highest ^226^Ra values were found in beef and cow’s milk compared to other farm animal products. This is because cattle can eat more grass than other farm animals. Although ^232^Th accumulates mainly on the stems and leaves of grasses, it was found that the concentration of ^232^Th was higher in animal products obtained from poultry fed by plants remaining in the soil after grazing of other animals^20^. Because the transfer factor from the ^232^Th radionuclide feed to the poultry products is at least 100 times greater than the transfer factors of the same radionuclide to other animal products.

Table 5 shows the comparison of the results obtained with other reports, the mean values of ^226^Ra in beef are higher than that those reported values, in Egypt, in United States, in Korea and in Taiwan^14,27–29^. In this study, the average value of ^40^K in beef is lower than the values reported in Korea by Choi *et al*. (2008), in Nigeria by Akinloye *et al*. (1999) and in Italy by Meli *et al*. (2013), while our obtained value is higher than the value reported by Harb *et al*. (2010) in Egypt^14,28,30,31^. Similarly, ^40^K concentrations in milk were higher than concentration in milk from other countries^13,14,28,32,33^.

**Table 5:** Comparison of values of ^226^Ra, ^232^Th and ^40^K (Bq kg^−1^) in animal product samples with reported values of other countries.

Animal Product	Country	Activity Concentration (Bqkg^−1^)			References
		^226^Ra	^232^Th	^40^K	
Beef	Turkey	1.9 × 10^−1^	2.8 × 10^−2^	99.6	This study
	Egypt	1.8 × 10^−2^	5.0 × 10^−4^	44.0	(^14^)
	United States	2.0 × 10^−2^	0.3–2.0		(^27^)
	Korea	3.6 × 10^−2^	1.45 × 10^−3^	90.1	(^28^)
	Nigeria	2.42		265.9	(^30^)
	Taiwan	1.2 × 10^−1^			(^29^)
	Italy			397.0	(^31^)
Poultry	Turkey	7.0 × 10^−2^	3.1 × 10^−2^	14.2	This study
	Egypt	2.7 × 10^−3^	4.9 × 10^−4^	2.81	(^14^)
	Korea	2.6 × 10^−2^	1.27 × 10^−3^	58.5	(^28^)
	Nigeria	2.11		280.9	(^30^)
	Taiwan	1.7 × 10^−1^			(^29^)
Cow Milk	Turkey	6.2 × 10^−1^	8.0 × 10^−3^	80.2	This study
	Egypt	3.3 × 10^−2^	8.0 × 10^−5^	21.4	(^14^)
	Syria			54.0	(^33^)
	Korea	2.2 × 10^−2^	8.0 × 10^−5^	52.9	(^28^)
	Iran			31.0	(^13^)
	Argentina			50.0–75.0	(^32^)

Table 5 reveals some differences in activity concentrations in animal products compared to the countries reported. This may be explained by physical properties of soil according to geographical location, the characteristics of the growing grass, climatic condition during the growth of the grass, the race of grazing animals and their spending time on pasture for grazing.

In this study, the annual consumption rate of animal products for people are obtained from Turkish Meat and Milk Institution’s report that are given in Table 6 ^34^. The radionuclide concentration values given in Table 4, the dose conversion factor for each radionuclide, and the annual consumption rate of animal products were used in Eq. 2 to evaluate the annual effective dose in the nutrients obtained from animals fed pasture-grass. Table 6 shows the calculated values of the average annual effective dose for ^226^Ra, ^232^Th and ^40^K in animal products. In the annual effective dose calculations here, the animals were assumed to eat dry-grass containing ^226^Ra or ^232^Th. For a more detailed calculation, the dose assessment should be made considering that animals also eat foods containing a number of daughter radionuclides and that these would have different transfer factors.

**Table 6:** The calculated annual effective dose values due to ^226^Ra, ^232^Th and ^40^K (Bq kg^−1^) radionuclides in animal products and average annual consumption by adult people of Kars region.

Products	Annual Consumption	Annual Effective Dose (µSv y^−1^)			
	(kg y^−1^)	^226^Ra	^232^Th	^40^K	Total
Beef	8.3	7.2 × 10^−2^	5.3 × 10^−2^	5.12	5.25
Mutton	4.1	2.9 × 10^−2^	4.5 × 10^−3^	0.35	0.38
Cow Milk	17.1	4.8 × 10^−1^	3.0 × 10^−2^	8.50	9.01
Sheep Milk	5.0	6.1 × 10^−2^	8.2 × 10^−4^	0.18	0.24
Poultry	17.9	5.2 × 10^−2^	1.3 × 10^−1^	1.57	1.76

The total annual dose due to internal irradiation caused by radiation emitted from the current ^226^Ra, ^232^Th and ^40^K radionuclides in the investigated animal products was evaluated as 16.6 µSv. The contribution of ^40^K radionuclide to total annual dose from animal products was 94.5%, while the contribution of ^226^Ra and ^232^Th radionuclides to the total annual dose was 4.2% and 1.3%, respectively.

There is no extra fertilization process in the pastures where the animals are grazed, but when the animals spend their times on pastures, they leave natural fertilizers rich in potassium to the pastures. Therefore, the highest contribution of ^40^K radionuclide to the total annual dose from animal products can be explained by the fact that soil properties support the mobilization of potassium and subsequent migration to the grass.

## Conclusions

Ingestion of contaminated foods is one of the routes of uptake of potentially dangerous radionuclides for man in particular due to importance in human diets. The activity concentrations of ^226^Ra, ^232^Th and ^40^K radionuclides were computed in food samples produced from animals consuming dry-grasses from 6 different grasslands of Kars region. The average annual effective dose from animal food consumption was figured out to be 16.6 µSv and the largest part of this dose was derived from the ^40^K natural radionuclide. As a result of this study, it has been determined that there will be no negative effects on human health and environment. Since the dairy products and meat of the animals growing in the region are consumed by people living in both the local and other cities of the country, systems should also be put in place to monitor radionuclides in animal products in order to reduce human exposure to radiation. Radiological assessment of environmental health risk can be done using known radioactivity values of environmental samples as in this study.
